# Mechanistic insights into nutrient profiles, cellulose, and hemicellulose dynamics in red and green *Toona sinensis* buds during cold storage

**DOI:** 10.3389/fpls.2025.1518924

**Published:** 2025-07-17

**Authors:** Guo-Fei Tan, Qian Zhao, Fu Wang, Shu-Yao Li, Zi-Yu Liu, Xin-Qi Zhang, Xiu-Lai Zhong, Shun-Hua Zhu, Xiu-Juan Lei, Zhong-Min Han, Jian Zhang

**Affiliations:** ^1^ Faculty of Agronomy, Jilin Agriculture University, Changchun, China; ^2^ Institute of Horticulture, Guizhou Academy of Agricultural Sciences/ Ministry of Agriculture and Rural Affairs Key Laboratory of Crop Genetic Resources and Germplasm Innovation in Karst Region, Guiyang, China; ^3^ College of Chinese Medicinal Materials, Jilin Agricultural University, Changchun, China; ^4^ Department of Biology, University of British Columbia, Okanagan, Kelowna, BC, Canada

**Keywords:** *T. sinensis* buds, nutrient component, cold storage, cytological observation, observation, gene expression

## Abstract

*Toona sinensis* (*T. sinensis*) is a popular woody vegetable with distinct red and green varieties in China. Despite its significance, research on the comparative nutrient composition profiles and cellulose and hemicellulose dynamics between these two varieties remains limited. This study comprehensively investigated the refrigerated storage characteristics of *T. sinensis* buds from multiple aspects, related to cellulose and hemicellulose synthesis. The results showed marked differences between the two varieties during 3 d postharvest storage. Green *T. sinensis* buds had more severe blackening at the petiole base. Green *T. sinensis* buds were also richer in vitamin C (Vc), protein, reducing sugars, flavonoids, and total phenols, while red *T. sinensis* buds had higher total sugar content. In terms of enzyme activities, red *T. sinensis* buds had elevated β-xylosidase metabolizes hemicellulose content over 28.65 mg·g^-1^ higher than that of green *T. sinensis* buds, while green *T. sinensis* buds increased cellulase (CL) activity led to a hemicellulose content 26.60 mg·g^-1^ high than red *T. sinensis* buds. The cell wall thickening and polygonal cell shape during storage were closely associated with the increase in hemicellulose content. Additionally, red *T. sinensis* buds exhibited elevated CAT and SOD activities in response to oxidative stress induced by increased MDA levels. In summary, green *T. sinensis* buds demonstrated higher nutritional value but reduced storage stability and enhanced lignification compared to red *T. sinensis* buds. This research not only provides a multi-dimensional understanding of *T. sinensis* storage characteristics, but also lays a foundation for the development of scientific storage and preservation methods.

## Introduction

*T. sinensis*, a perennial woody vegetable, has long been valued for its role as a functional foods and traditional medicinal materials, attributed to its distinctive flavor and rich nutrients in China (Yang et al., 2024; Zhao et al., 2023a). Its biological activities include antiviral, antioxidant, anti-inflammatory, and antidiabetic properties (Cao et al., 2024; Chen et al., 2007; Cheng et al., 2009; Peng et al., 2019; Wu et al., 2010; Zhang et al., 2014). Numerous *T. sinensis* varieties exist, primarily classified by the colors of their initial emergent spores and cotyledons. The color of new petioles and young leaves distinguishes two types: red *T. sinensis* and green *T. sinensis*. Red *T. sinensis* was characterized by its glossy appearance, robust flavor, crunchy texture, and high levels of water-soluble anthocyanins (Su et al., 2020). In contrast, green *T. sinensis* buds feature a mild flavor profile, lower oil content, and elevated dietary fiber levels (Zhai and Granvogl, 2020; Zhao et al., 2023b). Despite the potential to extend the harvesting period for *T. sinensis* buds beyond the Tomb Sweeping Day in China, driven by global warming and advancements in greenhouse cultivation, the market remains primarily centered on the spring season (Wang et al., 2023a; Zhao et al., 2021). Fresh *T. sinensis* buds contain approximately 80% water. Following harvest, cellular metabolism accelerates, resulting in shriveling, browning, and nutrient loss (Zhao et al., 2022). These changes accelerate senescence and result in a rapid decline in quality when stored at room temperature for 1 to 2 d (Lin et al., 2019). This poses a significant challenge to the sustainable development of the *T. sinensis* industry.

Interaction among cellulose and hemicellulose biosynthesis as a postharvest phenomenon occurring during storage of *T. sinensis* at room temperature or on the shelf (Zhang et al., 2024; Zhao et al., 2024). Cellulose, the primary structural component of plant cell walls, was a macromolecular polysaccharide composed of glucose units arranged linearly, providing mechanical strength to plant cells (Wolf-Rudiger and Markus, 2004). In contrast, hemicellulose comprises a heterogeneous group of polysaccharides with diverse structures. These polysaccharides intertwine with cellulose, forming a matrix that reinforces the integrity and rigidity of the cell wall (Ma et al., 2018). Both cellulose and hemicellulose serve as major sources of dietary fiber for humans (Mei et al., 2010). Their consumption positively influences intestinal peristalsis, glucose metabolism, and blood lipids regulation (Elleuch et al., 2011; Kaczmarczyk et al., 2012; Kaushik and Singh, 2011). However, during the postharvest storage of fruits and vegetables, environmental changes and physiological activities do not directly disrupt the biosynthesis of cellulose and hemicellulose. Instead, they indirectly trigger an imbalance in the synthesis process by interfering with the activities of enzymes involved in the synthesis of cellulose and hemicellulose (Cosgrove, 2024). This imbalance further leads to the abnormal accumulation of polysaccharides such as cellulose and hemicellulose in the cell wall, and affects the quality of fruits and vegetables and significantly shortens their storage life (Huang et al., 2019; Tan et al., 2019; Wang et al., 2020a; Wei et al., 2025; Wu et al., 2014; Xie et al., 2019; Zhang et al., 2021a).

In recent years, domestic academic research on *T. sinensis* buds has increased significantly, but there were few studies on the storage and preservation of *T. sinensis* buds in China (Liu et al., 2019; Wang et al., 2022b; Wang et al., 2023b; Xu et al., 2022). Considering the unique physiological characteristics of fresh *T. sinensis* buds, such as nutrient content, cellular structure, flavor components, research efforts have focused on developing effective processing and preservation techniques to extend market availability while preserving nutritional quality (Zhao et al., 2022). In terms of processing technologies, traditional and modern methods like freezing, salting, drying, and blanching have been extensively explored and applied (Zhang et al., 2024). Nevertheless, fully keeping the complete nutritional value and unique flavor of fresh *T. sinensis* buds remains challenging, and maintaining their nutritional value and flavor during physical storage is also a difficult problem for market demand (Wang et al., 2020b; Zhai and Granvogl, 2019; Zhang et al., 2022). However, considering various factors, such as cost-effectiveness, safety, operational feasibility, and environmental impact, cold storage, as a traditional method long applied in the postharvest storage and preservation of fruits and vegetables, remains an optimal choice to date. Although cold storage is a traditional approach, for some specific fruits and vegetables, take *T. sinensis* buds as an example. As a relatively rare vegetable with a high market price in spring, to ensure its good quality and commercial value after harvest, it is often necessary to make targeted adjustments and optimizations to the cold storage conditions and technical details according to its physiological characteristics, etc., so as to meet its unique preservation requirements (Shan et al., 2023; Zhang et al., 2021b). The use of cold storage has been shown to slow the respiration and metabolic rate of fruits and vegetables, inhibit microbial growth, and extend their shelf life (Romanazzi et al., 2017). The effectiveness of this technique has been demonstrated in various vegetables, including tomatoes (Yu et al., 2023, Yu et al., 2024), shiitake mushrooms (Hou et al., 2024; Xia et al., 2024), chili peppers (Mi et al., 2023), zucchini (Benitez et al., 2022; Zuo et al., 2022), water bamboo shoots (Qian et al., 2023).

To the best of our knowledge, certain progress has been achieved in applying cold storage to extend the shelf life of red *T. sinensis* buds. Nevertheless, research on the nutritional changes in both red and green *T. sinensis* during storage and the mechanisms of cellulose and hemicellulose are scarce. In light of this consideration, this study used red and green *T. sinensis* buds at equivalent developmental stage from Hongguang Village, Banqiao Town, Zijin County, Guizhou Province, China, to conduct storage experiments over a 4 d period. This was achieved through the application of cold storage techniques. The objective of this study was to systematically analyze dynamic changes in the physicochemical properties of *T. sinensis* buds before and after storage, focusing on four key aspects: appearance, nutrient content, cellular microstructure, and gene expression levels. The findings of this study advance our understanding of the cellulose and hemicellulose process between two varieties of *T. sinensis* buds during cold storage, offering valuable insights into their storage and preservation. This study delves deep into the analysis of changes in the appearance, nutrient content, cellular microstructure, and gene expression of red and green *T. sinensis* buds at a similar developmental stage. It further deepens the understanding of the cellulose and hemicellulose phenomenon and provides valuable information in terms of differences between *T. sinensis* varieties after cold storage.

## Materials and methods

### Plant materials and handling

In early April 2023, from the five-year-old red and the green *T. sinensis* tree in Hongguang Village, Banqiao Town, Zhijin County, Guizhou Province, China (105.71°E, 26.79°N), 600 g of red and 600 g of green *T. sinensis* buds (three sub-packages per sampling per group, with approximately 50 grams of samples in each bag), respectively, undamaged *T. sinensis* buds in optimal growth conditions, free from mechanical injuries, pests, and disease, were separately harvested. After harvesting, the surface dirt of *T. sinensis* buds was first washed away with distilled water, and the residual water was carefully blotted dry, and immediately stored at 4°C with dark environment. The first sampling was carried out at 7:00 a.m. on the first day of storage (0 d). Within the subsequent 3 d, sampling was conducted every 24 h, and for each sampling, three biological replicates were set for each sample. These sampled *T. sinensis* buds were then cut, homogenized, wrapped in foil, quickly frozen in liquid nitrogen, and stored at -80°C for total RNA isolation and the measurement of physiological and biochemical parameters.

### Appearance observation of *T. sinensis* buds

The initial colors of freshly harvested red and green *T. sinensis* leaves and petioles were documented through observation and photography. The buds were then stored at 4°C with dark environment, and their color changes, wilting, dehydration, shrinkage and decay were recorded daily for 3 d.

### Structure observation of *T. sinensis* buds

Freshly harvested leaves and petioles of red and green *T. sinensis* buds were promptly immersed in formalin-acetic acid-alcohol (FAA) fixative for at least 24 h. After fixation, the tissues were carefully trimmed to a flat configuration and then transferred to a dehydration cassette. The dehydrating cassette was placed in a dehydrator and underwent a well-defined gradient-based dehydration and wax-impregnation process: 75% ethanol for 4 h, 85% ethanol for 2 h, 90% ethanol for 2 h, 95% ethanol for 1 h, absolute ethanol I for 30 min, absolute ethanol II for 30 min, alcohol-zenzene for 5~10 min, xylene I for 5~10 min, xylene II for 5~10 min, and molten paraffin I, molten paraffin II, and molten paraffin III at 65°C each for 1 h. The subsequent experimental steps follow the research methods of predecessors in structure observation of *T. sinensis* buds (Zhao et al., 2024).

### Quality assessment

#### Reducing sugar, total sugar, protein, malondialdehyde, Vc content

Reducing sugar content was quantified via a colorimetric assay employing 3,5- dinitro salicylic acid (DNS). Approximately 0.2 g of *T. sinensis* buds were precisely weighed, followed by the addition of 2 mL of extraction solution. The mixture was homogenized in an ice bath, transferred to centrifuge tubes, and incubated in a water bath at 80°C for 40 min with intermittent oscillation every 4~5 min. The resulting solution was centrifuged, yielding a sample suitable for subsequent measurement. The test solution was combined with DNS reagent in the centrifuge tube and heated at 95°C for 5 min. The solution was rapidly cooled to room temperature, and absorbance was measured at 540 nm. A glucose standard curve was generated to calculate reducing sugar content. Results were expressed as micrograms per gram of fresh weight (µg·g^-1^, FW).

Total sugar content was determined using the following procedure: 0.1 g of *T. sinensis* buds was required. The buds were mixed with an appropriate volume of concentrated sulfuric acid aqueous solution (2:3 sulfuric acid to water) and ground into a homogeneous slurry. The slurry was subsequently incubated in a water bath at 95°C for 30 min. After incubation, 1 mL of NaOH and glycerol were added, followed by centrifugation to isolate the total sugar solution for analysis. To assess total sugar content, 0.14 mL of the total sugar test solution, distilled water, and DNS reagent were added to the centrifuge tube in a 2:2:3 ratio. After thorough mixing, the sample was heated at 95°C for 10 min to facilitate color development. After incubation, the sample was rapidly cooled to room temperature, 1 mL of distilled water was added, and the mixture was thoroughly vortexed. Absorbance was measured at 540 nm. Total sugar content was calculated from the glucose standard curve, with results expressed as mg·g^-1^, FW.

Protein content was determined following a series of analytical procedures. Following the methodology Caulmers, 0.1 g of *T. sinensis* buds was weighed, and an appropriate volume of distilled water was added. The buds were homogenized in an ice bath and subsequently centrifuged. The resulting supernatant was retained for protein quantification. The supernatant was combined with Caulmers Brilliant Blue G-250 solution at a 1:5 ratio and incubated at room temperature for 15 min to ensure complete dye-protein binding. 1 mL of the mixture was transferred to a glass cuvette and absorbance was measured at 595 nm. Total protein content was calculated from the standard curve, with results expressed as mg·g^-1^, FW.

MDA content was quantified using the thiobarbituric acid (TBA) assay. 0.1 g of *T. sinensis* buds was weighed, combined with an appropriate quantity of TCA extract, homogenized on ice, and subsequently centrifuged. The supernatant was collected and maintained on ice for immediate analysis. The test solution and TBA reagent were mixed at a 1:3 ratio and heated at 95°C for 30 min. The mixture was rapidly cooled in an ice bath and centrifuged immediately. Absorbance was measured at 532 nm and 600 nm, and MDA content was calculated using 1 mL of the supernatant in a 1 mL glass cuvette. Results were expressed as nmol·g^-1^, FW.

Vc content was determined using the 2,6-dichloroindophenol titration assay. 0.1 g of *T. sinensis* buds were weighed, 1 mL of 2% oxalic acid solution was added, and the mixture was homogenized on ice and centrifuged to extract the Vc solution for analysis. Titration was performed using an ascorbic acid standard solution, and a standard curve was generated to quantify the Vc content. Results were expressed as micrograms per gram of fresh weight (µg·g^-1^, FW).

#### Total phenolic and flavonoid content

The *T. sinensis* samples were pretreated as follows: They were dried in an oven at 80°C until reaching a constant weight. The dried samples were ground into fine powder using a mortar and pestle, and then sifted through a 40-mesh sieve to ensure particle uniformity. Precisely 0.1 g of *T. sinensis* bud powder was weighed and placed in a centrifuge tube. Subsequently, 2 mL of 60% ethanol solution was added, and the mixture was oscillated at 60°C for two hours to extract total phenols and flavonoids. After extraction, the supernatant was transferred to a clean centrifuge tube, and the final volume was adjusted to 2 mL with 60% ethanol to prepare the test solution for total phenol and flavonoid quantification.

Total phenol content was determined using the Folin-Ciocalteu colorimetric assay. A 0.05 mL aliquot of the test solution was added to a 2.0 mL centrifuge tube, followed by 0.25 mL of Folin-Ciocalteu reagent. The solution was thoroughly mixed and left to stand at room temperature for 2 min for initial color development. Next, 0.25 mL of sodium carbonate solution and 0.45 mL of distilled water were added to the tube. The solution was mixed well and left to stand at room temperature for 10 min to complete the color development. Absorbance at 760 nm was measured using a spectrophotometer, and the total phenolic content was calculated using the gallic acid standard curve. Results were expressed as milligrams per gram of dry weight (mg·g^-1^, DW).

Flavonoid content was quantified using the sodium nitrite-aluminum nitrate colorimetric assay. In a centrifuge tube, 0.5 mL of the sample solution and 0.3 mL of sodium nitrite solution were mixed thoroughly and left to stand at room temperature for 6 min. Then, 0.3 mL of aluminum chloride solution was added, thoroughly mixed and left to stand for 5 min. Following this, 0.4 mL of sodium hydroxide solution was added, the solution was mixed, and it was left at room temperature (25°C) for 15 min. Finally, absorbance was measured at 510 nm. A routine standard curve was used for quantitative flavonoid content, with results expressed as milligrams of flavonoids per gram of dry weight (mg·g^-1^, DW).

#### Cellulose and hemicellulose content

Cellulose content was quantified through an acid digestion protocol. The procedure involved weighing 0.1 g of dried *T. sinensis* bud powder into a centrifuge tube, followed by the addition of 10 mL of a 1:1 nitric acetic-acid mixture. The tube was subsequently placed in a boiling water bath for 30 min, with intermittent agitation 2 to 3 times. After heating, the sample was centrifuged, and the supernatant was discarded. The remaining precipitate, representing the crude cell wall material was retained. The precipitate was transferred to an ice-water bath, followed by the addition of 20 mL of 60% sulfuric acid. The mixture was left to digest overnight until complete dissolution of the precipitate. The solution was centrifuged, and the supernatant was transferred to a 100 mL volumetric flask. The volume was adjusted to 100 mL with 60% sulfuric acid, and the solution was further diluted 20-fold to prepare the cellulose solution for analysis. In a clean centrifuge tube, the test solution, anthrone, and concentrated sulfuric acid (4:1:10) were added sequentially. The mixture was thoroughly mixed and left to stand at room temperature for 15 min. Absorbance was measured at 620 nm, and cellulose content was calculated based on the cellulose standard regression equation. After acid treatment, hemicellulose was hydrolyzed into reducing sugars, which formed a reddish-brown complex with DNS reagent. This complex exhibited a characteristic absorption peak at 540 nm, and the hemicellulose content in *T. sinensis* buds was quantified based on the absorbance.

### Enzyme activity assay

The activities of superoxide dismutase (SOD, EC 1.15.1.1), peroxidase (POD, EC 1.11.1.7), polyphenol oxidase (PPO, EC 1.14.18.1), and catalase (CAT, EC 1.11.1.6) were determined following established and validated protocols. Specifically, SOD activity was measured using the nitro-blue tetrazolium (NBT) method, which is based on the ability of SOD to inhibit the photochemical reduction of NBT by superoxide radicals; One unit of SOD was defined as the amount of enzyme causing 50% inhibition of initial reduction of NBT under light at 560 nm (Chen et al., 2017). POD activity was measured via the guaiacol method, the reaction mixture (3 mL) consisted of 100 mM phosphate buffer (pH 6.0), 1.68 mL guaiacol, 1.14 mL H_2_O_2_ and 50 mL enzyme extract, the increase of absorbance at 470 nm was recorded continuously for 2 min to monitor the formation of tetraguaiacol, the extinction coefficient for tetraguaiacol was 26.6 L·mmol^-1^·cm^-1^ (Chen et al., 2017). PPO activity was determined using the catechol method, in which PPO catalyzes the oxidation of catechol to quinones, and the reaction progress is tracked by measuring the absorbance change; One unit of PPO activity is defined as the amount of enzyme required to cause a 0.01 change in absorbance at 525 nm per minute, per mg of tissue protein, in 1 mL of reaction system. CAT activity was quantified by via ultraviolet (UV) absorption spectroscopy, taking advantage of the characteristic absorption of hydrogen peroxide at a specific wavelength and the ability of CAT to decompose it; One unit of CAT activity is defined as the amount of enzyme required to the catalytic degradation of 1 μmol H_2_O_2_ per min per g of tissue. For the activities of the four aforementioned oxidases, along with (CL, EC 3.2.1.4), neutral xylanase (NEX, EC 3.2.1.8), and β-xylosidase (EC 3.2.1.37), the assays were conducted strictly in accordance with the detailed instructions provided in the kits procured from Suzhou Keming Biotechnology Co., Ltd. Enzyme activities in red and green *T. sinensis* buds during postharvest refrigeration were determined according to the manufacturer’s instructions provided in the respective kits. It is crucial to note that different enzymes exhibit distinct enzyme activity units; One enzyme activity unit is the amount of enzyme produced by decomposing 1 nmol of reducing sugar per minute per milliliter of liquid sample under the conditions of 50°C, and pH 6.0.

### Total RNA extraction and cDNA synthesis from *T. sinensis* buds

Total RNA was isolated from *T. sinensis* buds using a plant RNA extraction kit (GX, Beijing Huayueyang Biotechnology Co., Ltd., China) following the manufacturer’s protocol. RNA purity and concentrations were determined by measuring OD values using a microspectrophotometer (Nanodrop ND-100, Nanodrop Technology Inc., DE, USA). RNA integrity was verified through 1.5% agarose gel electrophoresis, after which the samples were stored at -80°C. RNA was reverse transcribed into cDNA using the HiScript III 1st Strand cDNA Synthesis Kit (+*g*DNA wiper) (Nanjing Novozymes Biotechnology Co., Ltd., China). Following the experimental protocol. The resulting cDNA was diluted 15-fold with sterilized ddH_2_O and stored at -20°C for future use.

### Expression analysis of genes related to cellulose and hemicellulose biosynthesis

To identify genes involved in cellulose and hemicellulose biosynthesis, the *T. sinensis* bud transcriptome database was analyzed using BioXM (2.7.1) software to identify open reading frames (ORFs). Gene sequences were further validated for accuracy and completeness via BLAST comparison using the NCBI database. Fluorescent primers specific to cellulose and hemicellulose biosynthesis genes were designed using Primer Premier 6 software. These primers were synthesized by Nanjing Kingsley Biotechnology Co., and the *TsActin* was used for reference gene (TABLE S1; Zhao et al., 2024).

### Statistical analysis

For data processing, the mean, and error analysis was calculated using WPS Office (Microsoft Excel 97-2003), the multiple comparisons analysis was calculated using one-way ANOVA (analysis of variance) test (the *p*-value of <0.05), and the while Origin 8.0 Software was utilized for graphing. For the correlation analysis, the Pearson’s correlation analysis method was adopted to quantify the linear relationship between variables, helping to understand the strength and direction of associations among different factors.

## Result

### Appearance observation of *T. sinensis* buds

**Figure 1:**
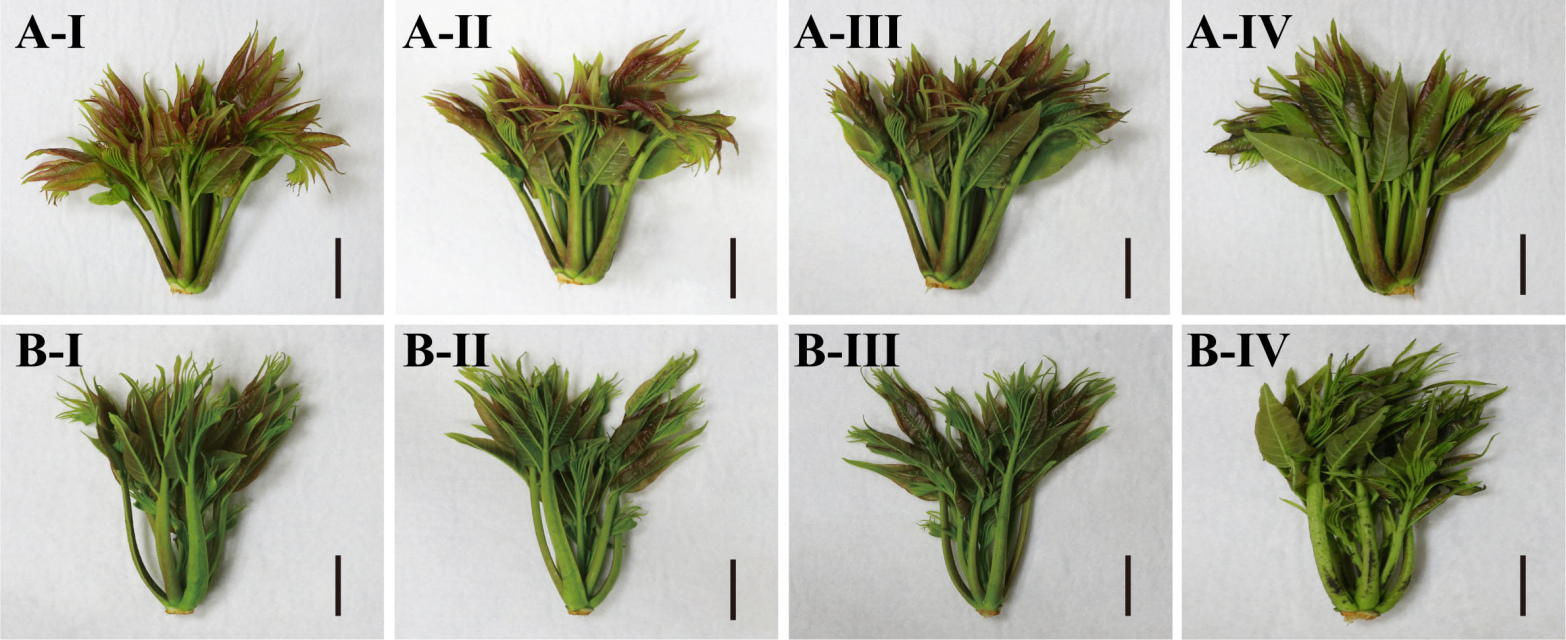
Appearance Changes of red and green *T. sinensis* buds during storage at 4°C. A-I. The red *T. sinensis* buds were stored at 4°C for 0 d. A-II. The red *T. sinensis* buds were stored at 4°C for 1 d. A-III. The red *T. sinensis* buds were stored at 4°C for 2 d. A-IV. The red *T. sinensis* buds were stored at 4°C for 3 d. B-I. The green *T. sinensis* buds were stored at 4°C for 0 d. B-II. The green *T. sinensis* buds were stored at 4°C for 1 d. B-III. The green *T. sinensis* buds were stored at 4°C for 2 d. B-IV. The green *T. sinensis* buds were stored at 4°C for 3 d. Bar = 2 cm.

To investigate the disparity in appearance quality between red and green *T. sinensis* at 4°C, we conducted 3 d observation of the phenotypic alterations in *T. sinensis* buds. Red *T. sinensis* buds displayed a vibrant color, with deep purplish-red leaves and petioles after harvest. Over the storage period, their appearance gradually eclipsed. After 2 d of storage, red *T. sinensis* buds showed a darkening of color and wilting of the leaves, indicating a loss of freshness. After 3 d of storage, decay started to develop at the tips of the leaves. The green *T. sinensis* buds, in contrast to the red ones, displayed a subtle reddish hue with noticeably lighter leaves green petioles. The changes during storage were more pronounced in the green *T. sinensis* buds. The leaves began to show signs of dehydration and shrinkage in 1 d. The large black spots appeared at the base of the petioles, along with significant decay at the leaf tips in 3 d (**Figure 1**). Red *T. sinensis* buds exhibited better appearance quality.

### MDA levels and antioxidant enzyme activities

**Figure 2:**
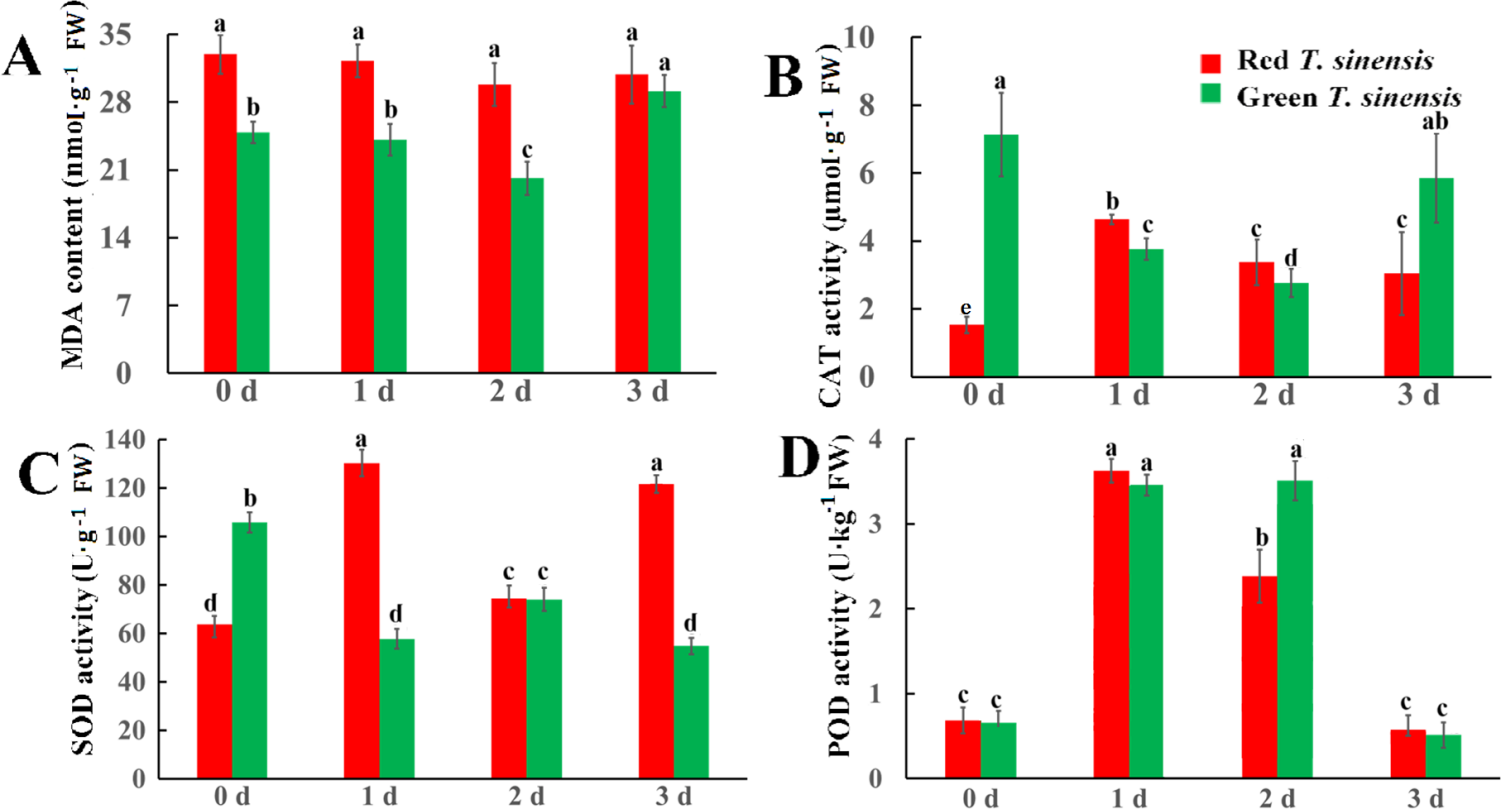
MDA levels and antioxidant enzymes activities of red and green *T. sinensis* buds during storage at 4°C. **(A)** The MDA level of red and green *T. sinensis* buds during storage at 4°C. **(B)** The CAT enzyme activities of red and green *T. sinensis* buds during storage at 4°C. **(C)** The SOD enzyme activities of red and green *T. sinensis* buds during storage at 4°C. **(D)** The POD enzyme activities of red and green *T. sinensis* buds during storage at 4°C. The use of distinct lowercase letters denotes statistically significant differences (*p*< 0.05) in relevant indices among the two *T. sinensis* cultivars across different storage times. The error bars represent the standard deviation (SD).

To evaluate the extent of membrane lipid peroxidation in postharvest *T. sinensis* at 4°C, we quantified the MDA content in both red and green *T. sinensis* buds during a 3 d storage period. Throughout the storage period, red *T. sinensis* buds exhibited consistently higher MDA levels than green *T. sinensis* buds. Notably, MDA level in red *T. sinensis* buds remained stable, while green *T. sinensis* buds displayed significant fluctuations. The most pronounced variation occurred in green *T. sinensis* buds, which showed decreased MDA level on 2 d of storage followed by a a sharp increase on 3 d, ultimately reaching 1.17-fold the baseline level (0 d). (**Figure 2A**). To investigate the role of the antioxidant system in modulating MDA accumulation in postharvest *T. sinensis*, we assessed the activity of three key antioxidant enzymes in both red and green *T. sinensis* buds, including CAT, SOD, and POD. At the start of storage (0 d), green *T. sinensis* buds exhibited significantly higher CAT and SOD activities compared to red *T. sinensis* buds, demonstrating 4.66-fold and 1.92-fold increase, respectively. However, the peroxidase (POD) activities of red and green *T. sinensis* buds were extremely similar to each other, nearly at the same level. Statistical tests showed no significant difference (*P* > 0.05) During storage, CAT and SOD activities in red *T. sinensis* buds peaked on 1 d, reaching 4.64 µmol·g^-1^ and 134.66 U·g^-1^, respectively. By 3 d, these activities had increased significantly relative to baseline (0 d), whereas green *T. sinensis* buds showed declines of 17.56% (CAT) and 47.89% (SOD). Notably, POD activities in both variants showed a dynamic trend of increasing and then decreasing, peaking at 3.69 U·kg^-1^ (red *T. sinensis* buds, 1 d) and 3.28 U·kg^-1^ (green *T. sinensis* buds, 2 d) before subsequent attenuation (**Figures 2B**–**D**).

### Vc, protein, total sugar, flavonoids and phenolic content

**Figure 3:**
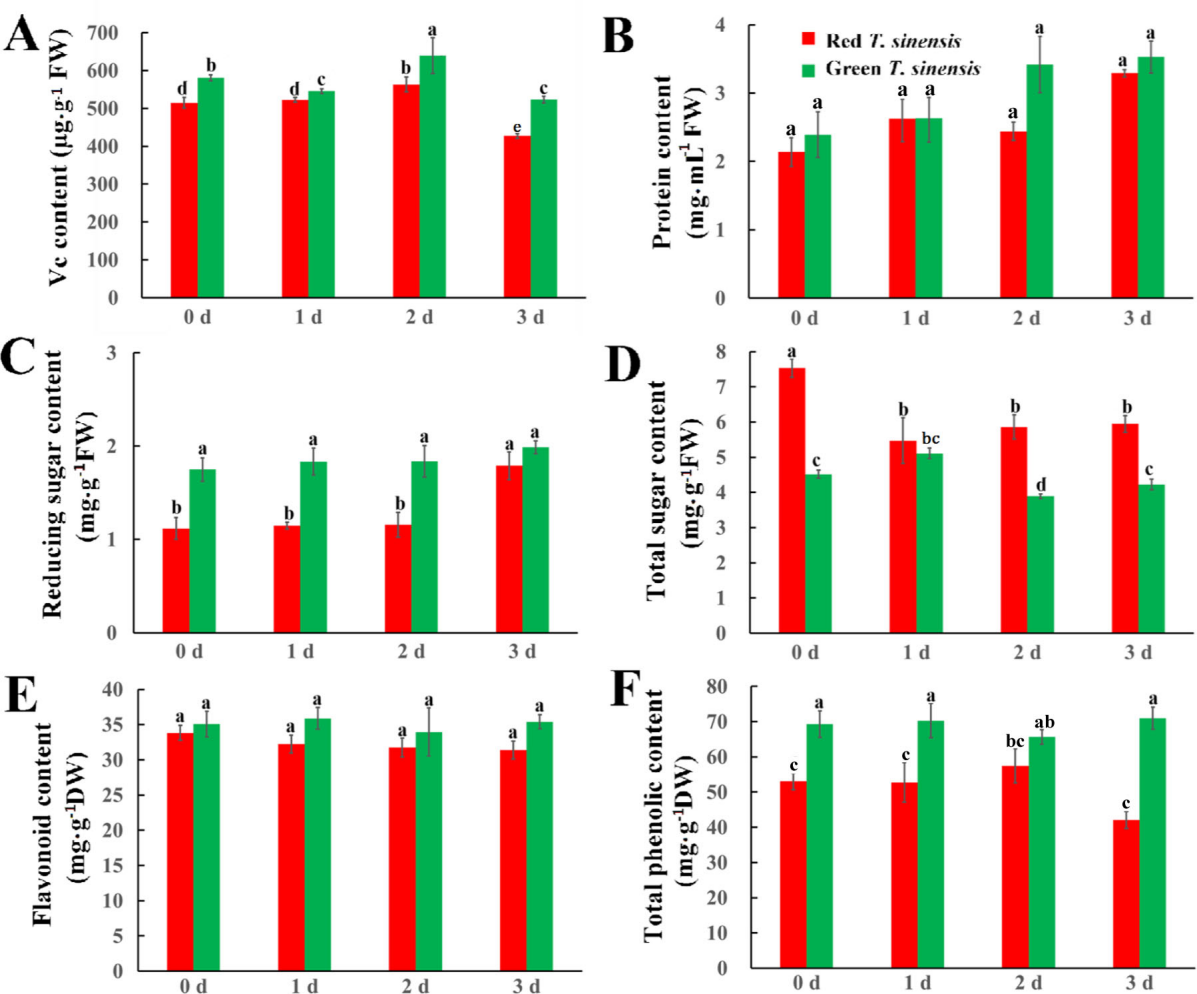
Dynamics of nutrients in red and green *T. sinensis* buds during storage at 4°C. **(A)** The Vc content of red and green *T. sinensis* buds during storage at 4°C. **(B)** The protein content of red and green *T. sinensis* buds during storage at 4°C. **(C)** The reducing sugar content of red and green *T. sinensis* buds during storage at 4°C. **(D)** The total sugar content of red and green *T. sinensis* buds during storage at 4°C. **(E)** The flavonoid content of red and green *T. sinensis* buds during storage at 4°C. **(F)** The total phenolic content of red and green *T. sinensis* buds during storage at 4°C. The use of distinct lowercase letters denotes statistically significant differences (*p*< 0.05) in relevant indices among the two *T. sinensis* cultivars across different storage times. The error bars represent the standard deviation (SD).

The changes in Vc and protein in red and green *T. sinensis* buds over the storage period were examined, the results revealed that green *T. sinensis* buds exhibited significantly higher Vc levels than red *T. sinensis* buds. By contrast, protein levels did not differ significantly between red and green *T. sinensis* buds throughout the storage period (*P* < 0.05). On 1 d of storage, Vc content in red *T. sinensis* buds was 504.85 µg·g^-1^, while protein content was 2.14 mg·mL^-1^. In contrast, green *T. sinensis* buds contained 587.46 µg·g^-1^ of Vc and 2.66 mg·mL^-1^ of protein. Compared to 0 d of storage, Vc levels in red and green *T. sinensis* buds decreased by 16.0% and 6.9% on 3 d of storage, respectively (**Figures 3A, B**). Conversely, protein content in *T. sinensis* buds increased over the storage period. Protein content increased by 54.0% in red *T. sinensis* buds and 43.0% in green *T. sinensis* buds compared to the start of the storage period (**Figure 3B**).

Sugar content and type not only influence the sweetness, flavor, and texture of fruits and vegetables, but also plays a crucial role in preserving the freshness of *T. sinensis* buds and reducing water loss. Significant differences were found in reducing and total sugar content, as well as their trends, between red and green *T. sinensis* buds. Green *T. sinensis* buds exhibited higher Reducing sugar content compared to red *T. sinensis* buds, whereas the total sugar content was lower. During storage, reducing sugar content in red *T. sinensis* buds increased significantly at 3 d of storage (*P* < 0.05). However, green *T. sinensis* buds exhibited no increase in reducing sugar content in throughout the storage period s (*P* > 0.05) (**Figure 3C**). Meanwhile, the total sugar content in red *T. sinensis* buds decreased significantly on 1 d of storage. Moreover, compared 3 d of storage with 0 d of storage, the total sugar content in red *T. sinensis* buds decreased by 1.68 mg·g^-1^, and in green *T. sinensis* buds decreased by 0.38 mg·g^-1^ (**Figure 3D**). The content levels of flavonoid and total phenolic in green *T. sinensis* buds were higher than red *T. sinensis* buds throughout the storage period. Additionally, the trends of these two variables demonstrated a striking inverse relationship. Specifically, the flavonoid and total phenolic content in green *T. sinensis* buds increased by 0.9% and 2.4%, respectively. Conversely, these compounds decreased by 7.3% and 1.6% in red *T. sinensis* buds. Notably, the changes in flavonoid and total phenolic levels were not statistically significant in either red or green *T. sinensis* buds throughout the storage period (**Figures 3E, F**).

### Cellulase-related enzyme activity

**Figure 4:**
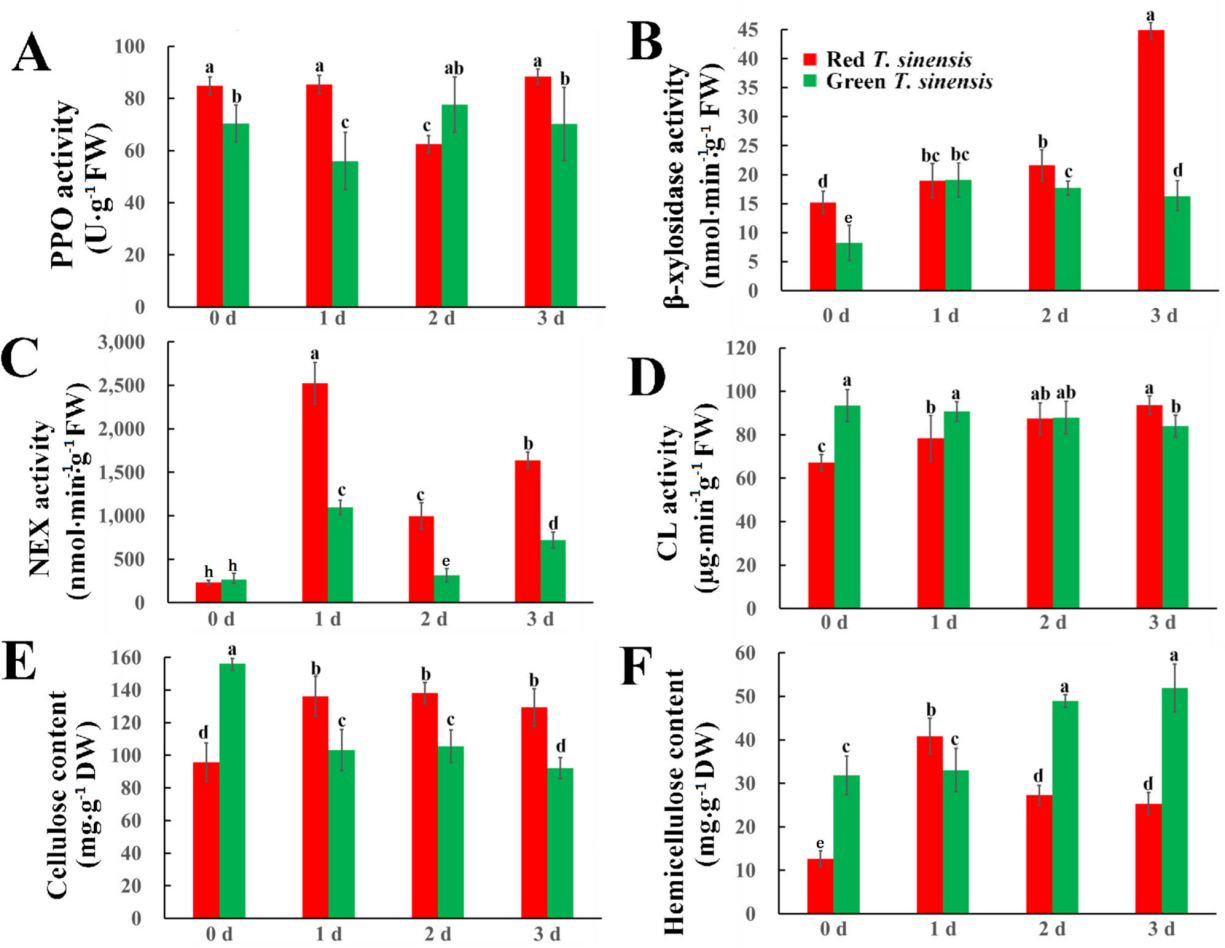
Cellulase enzyme activity and cellulose and hemicellulose contents in red and green *T. sinensis* buds during storage at 4°C. **(A)** The PPO enzyme activity of red and green *T. sinensis* buds during storage at 4°C. **(B)** The β-xylosidase enzyme activity of red and green *T. sinensis* buds during storage at 4°C. **(C)** The NEX enzyme activity of red and green *T. sinensis* buds during storage at 4°C. **(D)** The CL enzyme activity of red and green *T. sinensis* buds during storage at 4°C. **(E)** The cellulose content of red and green *T. sinensis* buds during storage at 4°C. **(F)** The hemicellulose content of red and green *T. sinensis* buds during storage at 4°C. The use of distinct lowercase letters denotes statistically significant differences (*p*< 0.05) in relevant indices among the two *T. sinensis* cultivars across different storage times. The error bars represent the standard deviation (SD).

Given the established correlation between cellulose and hemicellulose content in fruit quality during postharvest storage period, we quantified the cellulose and hemicellulose content, along with related enzymatic activities in both red and green *T. sinensis* buds. Significant differences were observed in the PPO enzyme activity changes among different *T. sinensis* species during postharvest storage. In red *T. sinensis* buds, PPO activity becomes lower on 2 d, and eventually reaches the initial level on 3 d. Green *T. sinensis* buds exhibited a decline-ascend-decline pattern throughout the storage period. After 3 d of storage, PPO enzyme activity in red *T. sinensis* buds was higher than that of green *T. sinensis* buds (**Figure 4A**).

β-xylosidase is a key enzyme responsible for degrading hemicellulose, a process that directly impacts the texture and firmness of fruits and vegetables. At the initial stage, β-xylosidase activity was higher in red *T. sinensis* buds than in green *T. sinensis* buds, with a significant difference of 6.98 nmol·min^-1^·g^-1^. During the storage period, distinct divergences were observed in the β-xylosidase activities of red and green *T. sinensis* buds. The β-xylosidase activity in red *T. sinensis* buds gradually increased. In contrast, that in green *T. sinensis* buds manifested as an initial upswing followed by a downward trend. Notwithstanding these disparate trends, when compared with the 0 d of storage, on the 3 d of storage, the β-xylosidase activity in red *T. sinensis* buds experienced a remarkable increase, reaching nearly two-fold in the initial level, while the β-xylosidase activity in green *T. sinensis* buds significantly doubled (**Figure 4B**).

The NEX enzyme is involved in the degradation of xylanase within the cell walls of fruits and vegetables, a process that weakens the cell walls and softens their texture. The NEX enzyme activity in red and green *T. sinensis* buds peaked at 2,526 and 1,095 nmol·min^-1^·g^-1^ after 1 d and 2 d of storage. The most significant fluctuation in NEX enzyme activity during storage was observed in two types of *T. sinensis* buds. After 3 d of storage, NEX enzyme activity in red and green *T. sinensis* buds significantly increased by 431.91% and 321.15%, respectively (**Figure 4C**).

Unlike β-xylosidase, CL primarily facilitates cellulose degradation, acting as a critical marker of fruit and vegetable aging. At 0 d and 1 d of storage, the ratio of CL activity in green *T. sinensis* buds to that in red *T. sinensis* buds was 1.4 and 1, respectively. After 2 d storage, CL activity in both red and green *T. sinensis* buds was nearly identical. After 3 d of storage, CL activity in red *T. sinensis* buds was significantly higher compared to green *T. sinensis* buds. Compared to the initial storage period, CL activities increased by 39.45% in red *T. sinensis* buds and decreased by 10.13% in green *T. sinensis* after 3 d of storage (**Figure 4D**). Significant differences were observed in the cellulose and hemicellulose levels between green and red *T. sinensis* buds. At 0 d of storage, green *T. sinensis* buds contained 1.65-fold higher cellulose and 1.99-fold greater hemicellulose than red ones. During storage (1**–**3 d), cellulose dynamics diverged markedly between two types of *T. sinensis* buds: red *T. sinensis* buds accumulated 40.46 mg·g^-1^ more cellulose relative to baseline, whereas green *T. sinensis* buds showed of 53.16 mg·g^-1^ reduction (**Figure 4E**). Hemicellulose content in green *T. sinensis* buds consistently exceeded red buds throughout storage, except on 2 d. Peak values occurred asynchronously: red *T. sinensis* buds reached maximal hemicellulose (41.92 mg·g^-1^) on 1 d of storage, whereas green buds peaked later (54.04 mg·g^-1^) on 3 d of storage. Both phenotypes exhibited significant hemicellulose accumulation by 3 d compared to baseline (0 d) (**Figure 4F**).

### Organizational structure observation

**Figure 5:**
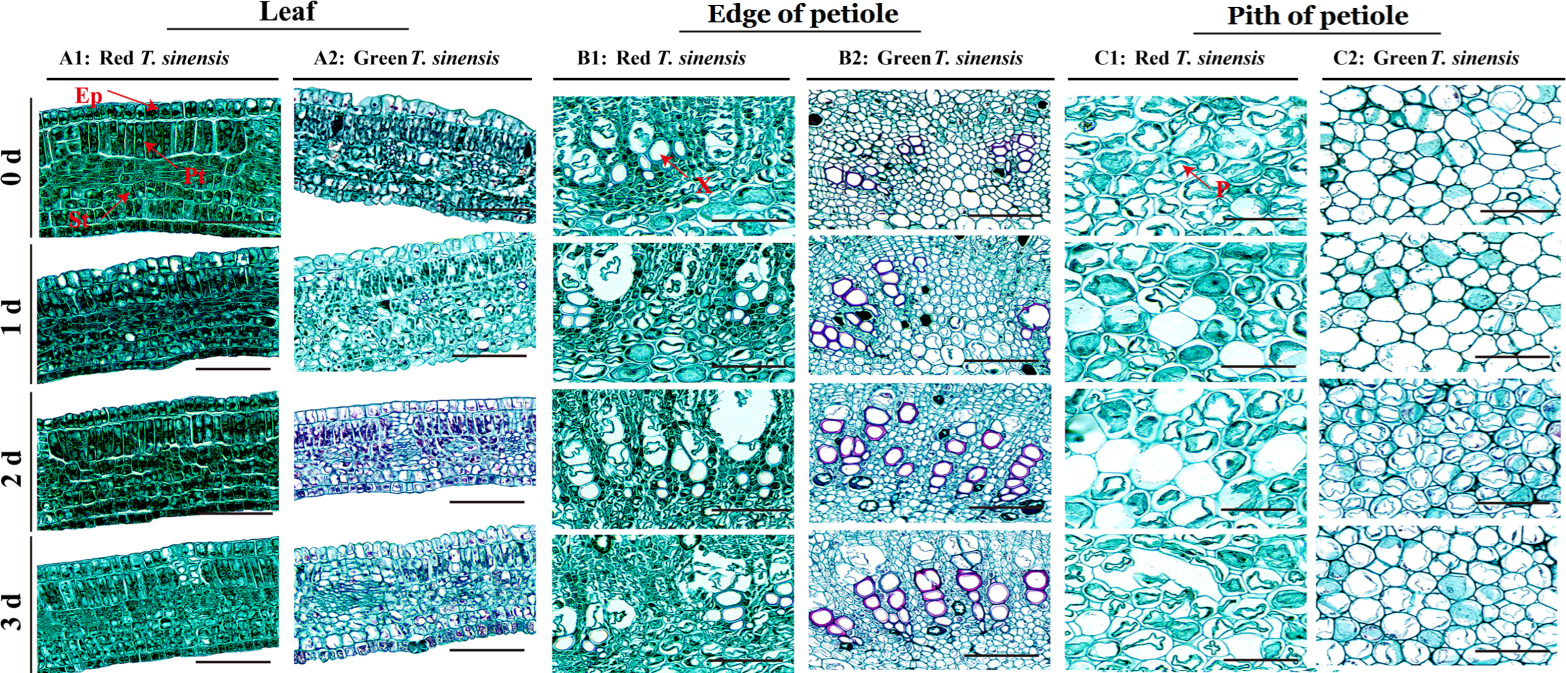
Leaf and petiole structure of red and green *T. sinensis* buds during storage at 4°C. **A1**: Leaf structure of red *T. sinensis* buds; **A2**: Leaf structure of green *T. sinensis* buds; **B1**: Edge of petiole structure of red *T. sinensis* buds; **B2**: Edge of petiole structure of green *T. sinensis* buds; **C1**: Pith of petiole structure of red *T. sinensis* buds; **C2**: Pith of petiole structure of green *T. sinensis* buds. Ep: epidermis; Pt: palisade tissues; St: spongy tissues; X: xylem; P: pith. Bar =100 μm.

The palisade tissue (Pt) cells of the red *T. sinensis* buds initially exhibited a long-columnar morphology, organized in a regular, compact structure. With prolonged storage, a pronounced morphological transformation was observed in the Pt cells of red *T. sinensis* buds. Initially, the cells maintained their long columnar shape, but gradually transitioned to an irregular configuration. Additionally, the cell edges developed a wrinkle-like appearance, which became increasingly pronounced after 3 d of storage. After 2 d of storage, the spongy tissue cells in red *T. sinensis* buds maintained a regular arrangement. Over time, these cells showed gradual swelling and a subsequent disruption in their regular arrangement by the 3 d of storage. In contrast to red *T. sinensis* buds, the palisade and spongy tissue cells of green *T. sinensis* buds displayed an initially irregular distribution throughout the leaves from the onset of storage (**Figures 5A1, A2**).

Regarding the edge of petiole xylem cells, the red *T. sinensis* buds were observed to exhibit a more rounded morphology at the start of storage. In contrast, the xylem cells at the edge of petiole of green *T. sinensis* buds exhibited a distinctly right-angled configuration. Green *T. sinensis* buds were founded to have a higher number of petiole xylem cells compared to red *T. sinensis* buds under the same storage conditions. As the storage period progressed, the degree of lignification in the edge of petiole xylem cells of both red and green *T. sinensis* buds increased. After being stained with safranin, which can color lignin red, it was observed that the red color in the edge of petiole xylem cells of green *T. sinensis* buds was deeper than that of red *T. sinensis* buds. Moreover, with the increase of the storage time, the red-colored area gradually expanded, and the color deepened. This clearly suggests that the lignin content of *T. sinensis* buds increases with the extension of the storage period. Notably, the green *T. sinensis* buds exhibited a more pronounced alteration. Specifically, the petiole xylem cells the red *T. sinensis* buds largely retained their spherical shape, displaying only a marginal enhancement in both cell wall color and thickness. By contrast, the petiole xylem cells in green *T. sinensis* buds showed a progressive intensification in cell wall pigmentation and adopted a distinctly organized polyhedral structure throughout the cold storage phase (**Figures 5B1, B2**).

The postharvest refrigeration process similarly induced substantial changes in the pith of petiole vesicle cells of both red and green *T. sinensis* buds. In red *T. sinensis* buds, the petiole pith cells appeared loosely arranged in an irregular pattern. Despite this, the intracellular vesicles in these cells remained relatively full. However, as storage time progressed, this pith of petiole vesicle cells sustained damage and ruptured, releasing their intracellular contents. This phenomenon was evident from the 1 d of postharvest storage and exhibited a gradual intensification over the storage period. In contrast to the changes seen in red *T. sinensis* buds, the pith of petiole vesicle cells of green *T. sinensis* buds remained a dense, regular arrangement, with the pith of petiole vesicle cells remained full throughout the postharvest cold storage period. These findings offer a vital reference point for elucidating the structural changes in different *T. sinensis* buds during cold storage (**Figures 5C1, C2**).

### Cellulose and hemicellulose related gene expression level

**Figure 6:**
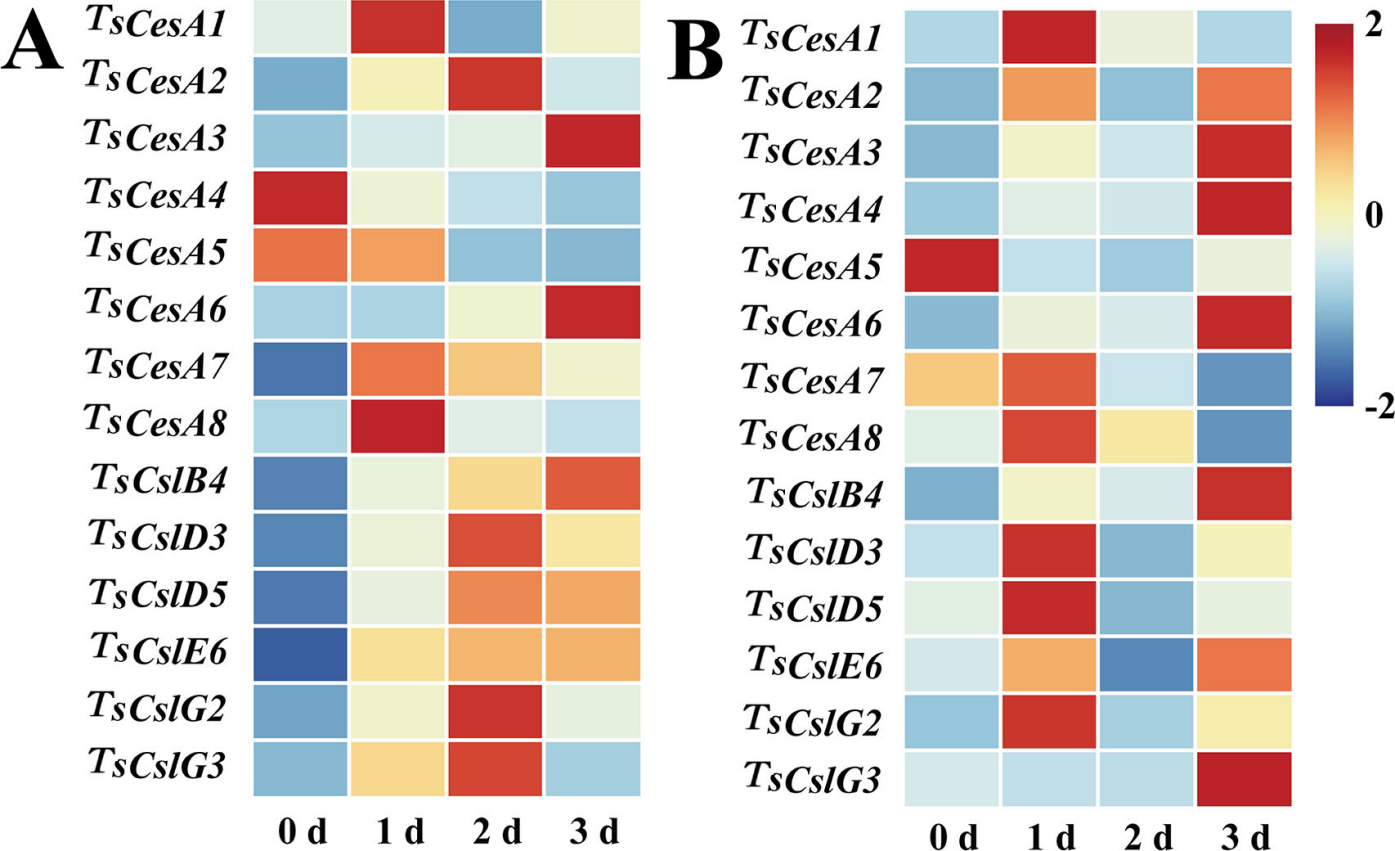
Cellulose-related gene expression levels in red and green *T. sinensis* buds during storage at 4°C. **(A)** Cellulose-related gene expression levels in red *T. sinensis* buds during storage at 4°C. **(B)** Cellulose-related gene expression levels in green *T. sinensis* buds during storage at 4°C. The correlation analysis in parallel with heat map construction using the Chiplot online platform, applying Pearson’s correlation coefficient for measuring variable relationships and employing the Standard Scaler algorithm for data normalization.

Total of 8 cellulose synthase genes (*TsCesA1*, *TsCesA2*, *TsCesA3*, *TsCesA4*, *TsCesA5*, *TsCesA6*, *TsCesA7*, and *TsCesA8*), along with 6 cellulose-like synthase genes (*TsCslB4*, *TsCslD3*, *TsCslD5*, *TsCslE6*, *TsCslG2*, and *TsCslG3*), were identified from the transcriptome database of *T. sinensis* buds (Zhao et al., 2024). The expression profiles of these genes were evaluated during cold storage of red and green *T. sinensis* buds using qRT-PCR. The results revealed substantial variations in the expression of cellulose-related genes across distinct stages of *T. sinensis* bud storage. For red *T. sinensis* buds, most cellulose-like synthase genes showed elevated expression levels in the later stages of postharvest storage, specifically at 2 and 3 d. Notably, the expression levels of certain cellulose synthase genes, such as *TsCesA2*, *TsCesA3*, and *TsCesA6*, were significantly upregulated during the late storage phase compared to earlier periods. In green *T. sinensis* buds, with the exception of *TsCesA5*, the expression levels of both cellulose and cellulose-like synthase genes were increased at 1 and 3 d of storage (**Figures 6A, B**).

**Figure 7:**
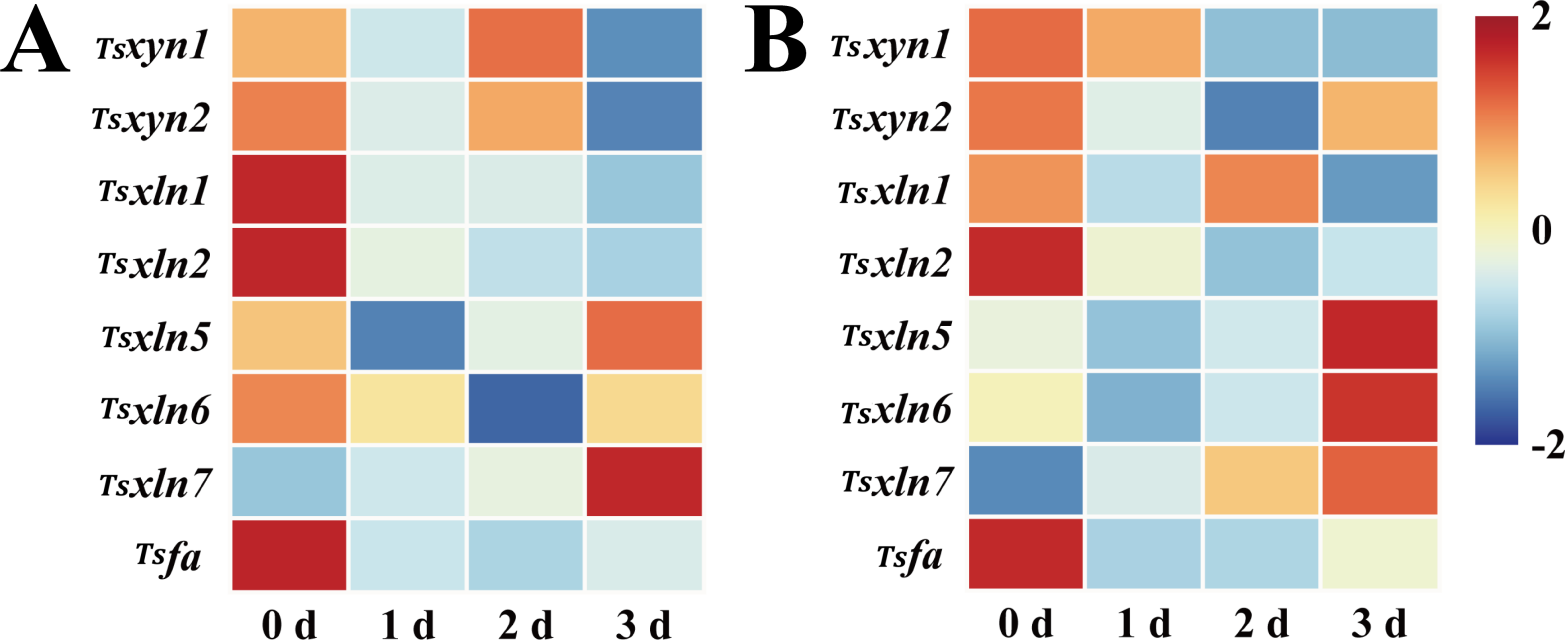
Hemicellulose-related gene expression levels in red and green *T. sinensis* buds during storage at 4°C. **(A)** Hemicellulose-related gene expression levels in red *T. sinensis* buds during storage at 4°C. **(B)** Hemicellulose-related gene expression levels in green *T. sinensis* buds during storage at 4°C. The correlation analysis in parallel with heat map construction using the Chiplot online platform, applying Pearson’s correlation coefficient for measuring variable relationships and employing the Standard Scaler algorithm for data normalization.

The expression profiles of *Tsxyn1* and *Tsxyn2* in red *T. sinensis* buds during cold storage presented a dynamic trend. Initially, their expression decreased, then increased, and finally declined. *Tsxyn1* expression peaked on the 2 d storage. For *Tsxyn2*, its expression was highest at the beginning of storage and declined significantly as the storage period went on. Regarding *Tsxln1*, *Tsxln2*, and *Tsfa*, their expression reached the peak at 0 d of storage and then gradually declined during the storage process. In contrast, *Tsxln7* expression increased steadily throughout the storage period, reaching its maximum on the 3 d. *Tsxln5* and *Tsxln6* exhibited similar expression patterns, with an initial decrease followed by an increase. Notably, the expression of *Tsxln5* on the 3 d was markedly higher than at the start of storage, while *Tsxln6* showed a decline at the same time point (**Figure 7A**).

The expression of hemicellulose-related genes in green *T. sinensis* buds closely resembled the pattern observed in red *T. sinensis* buds. Most of these genes demonstrated elevated expression levels at both 0 d and 3 d of storage. This clearly indicates that the expression of hemicellulose genes was predominantly concentrated at these two time points. These findings highlight significant temporal and spatial variations in the expression of cellulose and hemicellulose synthase genes during cold storage of *T. sinensis* buds (**Figure 7B**).

### Correlation analysis

**Figure 8:**
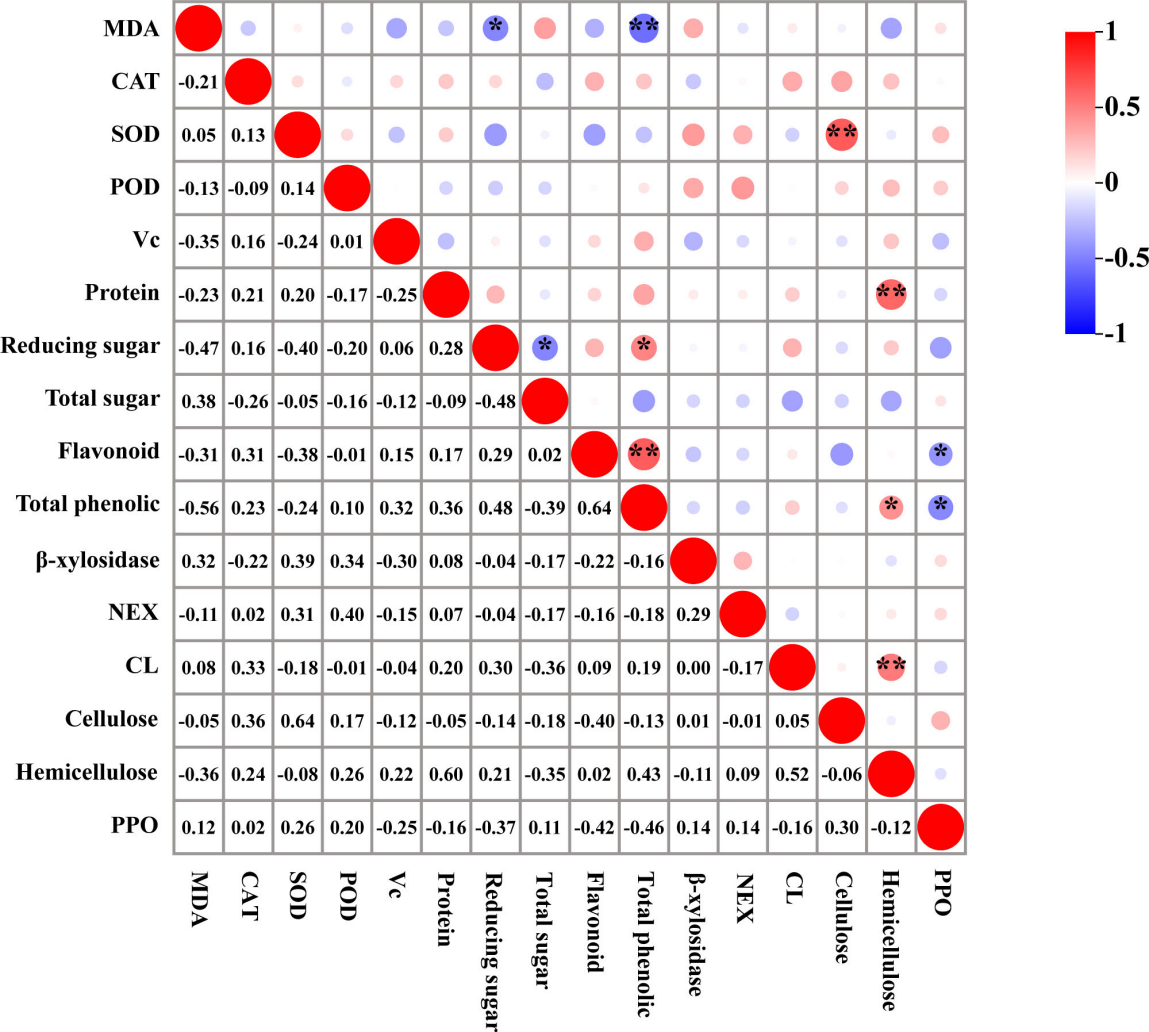
Correlation analysis of physiological indexes of *T. sinensis* buds during storage at 4°C. *: was significant correlation (0.05); **: was highly significant correlation (0.01).

The correlation analysis of physiological indices during post-harvest cold storage of *T. sinensis* buds revealed a significant positive correlation between reducing sugar content and total phenol content (0.48*). Furthermore, a notable positive correlation was identified between total phenol content and hemicellulose content (0.43*). A highly significant positive correlation was detected between SOD and cellulose content (0.64**), protein and hemicellulose content (0.60**), flavonoid and total phenol content (0.64**), and CL and hemicellulose content (0.52**). Regarding negative correlations, a significant inverse relationship was identified between MDA content and reducing sugar content (-0.47*), and between reducing sugar and total sugar content (-0.48*). Additionally, a significant negative correlation was observed between flavonoid content and PPO content (-0.42*), as well as between total phenolics and PPO content (-0.46*). Moreover, the correlation between MDA content and total phenolics content was highly significant (-0.56**) (**Figure 8**).

## Discussion

The visual appearance and color of fruits and vegetables are widely recognized as primary indicators of freshness by consumers (Chen et al., 2018). In this study, identical conditions were applied for the postharvest storage of red and green *T. sinensis* buds over a 3 d period. A comparative analysis of the color changes between the two varieties revealed that both red and green *T. sinensis* buds exhibited wilting, decay, signs of dehydration and shrinkage. Notably, the changes were more pronounced in the green *T. sinensis* buds. This phenomenon suggests that red *T. sinensis* buds possess a superior capacity for water retention, likely attributable to the cell structure of the leaves (Ghanem et al., 2012). Specifically, the palisade and spongy tissues cells in the leaves of red *T. sinensis* buds were densely packed and regularly arranged, despite morphological changes occurring during storage. By contrast, the cells of the palisade and spongy tissues cells in the leaves of green *T. sinensis* buds were consistently irregularly in their arrangement. Additionally, green *T. sinensis* buds developed substantial black spots at the edge and pith of petiole base after 3 d of storage. Increased enzyme activity leads to the breakdown of cell walls, and tissue softening, creating a favorable environment for microbial invasion. Subsequent microbial invasion resulted in tissue necrosis and the eventual formation of black spots (Ren et al., 2020).

MDA serves as a critical indicator of oxidative stress, and its concentration indirectly reflects the antioxidant capacity of organisms during postharvest storage of fruits and vegetables (Zhang et al., 2018). At the beginning of the storage period, red *T. sinensis* buds exhibited significantly higher MDA levels than green *T. sinensis* buds, which may be attributed to the relatively higher oxidative stress experienced by red *T. sinensis* buds during normal growth (Ackah et al., 2022). Consequently, the antioxidant responses of red and green *T. sinensis* buds followed different patterns during postharvest storage. Green *T. sinensis* buds exhibited CAT and SOD enzyme activities at the onset of storage, indicating a robust antioxidant capacity during the early stage to mitigate oxidative stress encountered postharvest. However, after 3 d of storage, the MDA level in green *T. sinensis* buds significantly increased compared to 0 d, while CAT and SOD enzyme activities declined, and POD enzyme activity remained largely unchanged. This suggests that the antioxidant system in green *T. sinensis* buds was progressively impaired or unable to effectively sustain its antioxidant capacity during storage (Zhang et al., 2019). In contrast, despite the initially high MDA level, red *T. sinensis* buds showed a slight decrease in MDA during storage, while CAT and SOD activities increased and POD activity remained relatively stable. These findings imply that red *T. sinensis* buds possess a more stable and effective antioxidant mechanism, enabling them to better cope with oxidative stress during postharvest storage.

This study demonstrated that green *T. sinensis* buds consistently contained higher levels of Vc, reducing sugar, flavonoid and total phenol than red *T. sinensis* buds throughout storage. In contrast, red *T. sinensis* buds displayed higher total sugar content. This discrepancy likely stems from the genetic traits of *T. sinensis* buds, which contribute to their distinctive flavor and different taste profile (Wang et al., 2022b). Notable differences emerged in the nutrient profiles of red and green *T. sinensis* buds throughout postharvest storage. Compared to 0 d, only protein and reducing sugar content in red *T. sinensis* buds increased after 3 d of storage. In contrast, only the Vc content in green *T. sinensis* buds showed a significant decrease, and the total sugar content was no significant change, while other components increased. During *T. sinensis* bud storage, the decline of Vc may result from oxidation reaction, whereas the increase in other nutrients could be due to the decomposition and transformation of various substances (Gil et al., 2006; Mostafidi et al., 2020).

PPO, β-xylosidase and NEX enzyme activities in red *T. sinensis* buds were higher than those in green *T. sinensis* buds during storage, whereas green *T. sinensis* buds showed greater CL activity. These enzyme activity modulations directly influenced cellulose and hemicellulose content in *T. sinensis* buds (Peres et al., 2017). Elevated β-xylosidase activity in *T. sinensis* buds accelerated hemicellulose degradation, resulting in a reduced hemicellulose content. Increased CL activity in *T. sinensis* buds promoted cellulose degradation, thereby lowering cellulose content (Chen et al., 2023; Jia et al., 2021). Safranin staining solution has the ability to impart a red color to lignin. Notably, there exists a positive correlation such that the higher the lignin content, the more intense the red hue. Through subjective assessment, upon light microscopic examination of paraffin sections, we discerned that the cell walls of the xylem cells in the petioles of green *T. sinensis* buds exhibited a progressively darker coloration, and the cells assumed a regular polyhedral configuration, which strongly implies an elevated degree of lignification (Cai et al., 2006; Huang et al., 2019). During *T. sinensis* bud storage, increased cellulase activity promoted cellulose deposition, thickening the cell wall, enhancing mechanical strength and maintaining the polyhedral cell shape (Wang et al., 2023c).

Cellulose and hemicellulose gene expression levels interact with their contents, forming a complex regulatory network (Rao et al., 2019). During postharvest storage of *T. sinensis* buds, only *TsCesA3*, *TsCesA6*, and *TsCslB4* genes expression levels gradually increased over time, mirroring the cellulose content trend, suggesting these genes play a positive regulatory role in cellulose synthesis. In green *T. sinensis* buds, the majority of cellulose genes were significantly expressed on 1 and 3 d of storage, with *TsCesA3*, *TsCesA4*, *TsCesA6*, *TsCslB4*, and *TsCslG3* showing notable expression increases at 3 d of storage. However, cellulose content in *T. sinensis* buds trended downward on 1 and 3 d of storage, implying an inverse correlation between cellulose content. During postharvest storage, more than half of the genes exhibited significant expression on 0 and 3 d of storage, with *Tsxln7* gene expression level in red and green *T. sinensis* buds increasing markedly on 3 d of storage. These results suggest that *Tsxln7* gene may be a key regulator of hemicellulose synthesis and accumulation during *T. sinensis* bud storage.

## Conclusion

This study showed that red *T. sinensis* buds exhibit greater storage resilience and a more stable antioxidant system than green buds, which maintained higher levels of Vc, protein, reducing sugars, flavonoids, and total phenols. Red *T. sinensis* buds retained more total sugar, green *T. sinensis* buds exhibited a pronounced accumulating of hemicellulose. The xylem cell walls of green *T. sinensis* buds were observed to display a notably deeper red hue compared to those of red *T. sinensis* buds. These findings offer insights into the storage characteristics of *T. sinensis* buds, providing a foundation for improved preservation strategies.

## Data Availability

The original contributions presented in the study are publicly available. This data can be found here: NCBI, PRJNA1289419.
