# Correction: Mechanistic insights into nutrient profiles, cellulose, and hemicellulose dynamics in red and green *Toona sinensis* buds during cold storage

**DOI:** 10.3389/fpls.2025.1668927

**Published:** 2025-07-30

**Authors:** Frontiers Production Office

**Affiliations:** Frontiers Media SA, Lausanne, Switzerland

**Keywords:** *T. sinensis* buds, nutrient component, cold storage, cytological observation, observation, gene expression

The affiliation for Xiu-Juan Lei was incorrectly assigned.

Affiliation 3, “College of Chinese Medicinal Materials, Jilin Agricultural University, Changchun, China” was omitted for author Xiu-Juan Lei.

This affiliation has now been added for author Xiu-Juan Lei.

Author Xiu-Juan Lei was erroneously assigned to affiliation 2 “Institute of Horticulture, Guizhou Academy of Agricultural Sciences/Ministry of Agriculture and Rural Affairs Key Laboratory of Crop Genetic Resources and Germplasm Innovation in Karst Region, Guiyang, China.”

This affiliation has now been removed for author Xiu-Juan Lei.

The original version of this article has been updated.

