# Supplementary material for: Mechanistic insights into nutrient profiles, cellulose, and hemicellulose dynamics in red and green Toona sinensis buds during cold storage
Source: Front Plant Sci. 2025 Jul 17;16:1518924. doi: 10.3389/fpls.2025.1518924 (PMC12344525; doi:10.3389/fpls.2025.1518924)
Supplement: Supplementary file 1 [file Table1.docx]

**TABLE S1** Primer for RT-qPCR related to cellulose and hemicellulose synthesis used in this study

| **Gene** | **Forward Primer (5’-3’)** | **Reverse Primer (5’-3’)** | **Substance** |
| --- | --- | --- | --- |
| *TsCesA1* | TCTATACTTGCTGTGGATTACCC | CTCTAGGCTCTATATTGTGCTTCT | Cellulose |
| *TsCesA2* | GAGTGGAGTAAGCATCGAAGAAT | GTCGGAGGAATTAGAAGAGTTGT | Cellulose |
| *TsCesA3* | TCCAATCACTGCTATTCCTCTTC | CCACCATTCATCAATTCCAACAC | Cellulose |
| *TsCesA4* | CAGGAAGCGTTAATGGTAAGGAT | CGGAGGATAATGACTATGCGATAT | Cellulose |
| *TsCesA5* | GTGGTTCTGACTCCGATTTACAT | AGCAACACTTCCATAGCCATAAG | Cellulose |
| *TsCesA6* | GTTGGTATTGATGAATGGTGGAG | ACTATAAGCAGTGTTGTTGGAGG | Cellulose |
| *TsCesA7* | GAAGGAGATGACGATGAAGAAGA | GCTATAACTGGTGGGAATTGAGA | Cellulose |
| *TsCesA8* | TTATGGTCTATGGCTCACTTCTG | CTTTCAATGGGTCAACTGTACTC | Cellulose |
| *TsCslB4* | GCCGATGATGAGTTACTAAAGAAG | GAGCCATATAACCAGCCAACATT | Cellulose |
| *TsCslD3* | CATTCACCTCCATATTCCTCATTG | CTCATTCCTCCACCATTCTTCTA | Cellulose |
| *TsCslD5* | AGGATTCAATTCAGTGACGAGAG | CATTATCTCCATCGCTAACCTCT | Cellulose |
| *TsCslE6* | TGTAAGACAGACTGGAAGAGAAC | CCTCCGCATTGTATCAATAATCC | Cellulose |
| *TsCslG2* | CAAAGTTCTGCCCTCCTATTTATG | GTGATGATAACCTCCTCCAAGTG | Cellulose |
| *TsCslG3* | CTAACTATGTCGGAACTGGAACC | CTCTGCCAACGAACCATATCTAA | Cellulose |
| *Tsxyn1* | GGCTTGGGTTCTAAATCTTACAG | GAGTTGCTAATGGATCTGATTGG | Hemicellulose |
| *Tsxyn2* | GATAAGATTCGTAAGCGTGATGTC | TCTGTCCAATACCACTTCAACTC | Hemicellulose |
| *Tsxln1* | GTGACTCTGTTGGTGTTCTATATG | GCCTCATCTGGACTGTAATAGTAT | Hemicellulose |
| *Tsxln2* | GTGTTATGTGCTCGTACAATCAG | GGTGTTGAGGTATAATGTTGAGTG | Hemicellulose |
| *Tsxln5* | CATGGTATCCGCAAGAGTATGTA | TTGGAGAATGATGAGTAGCTTAGG | Hemicellulose |
| *Tsxln6* | CTTCCTATGACTTGGTATCCTGAG | GAACTGGTAACTGTAATTGGTGTAG | Hemicellulose |
| *Tsxln7* | TCATCTATGATGCTCAAGGATACG | TAACAGAGAAGAGGTTGTGAAGG | Hemicellulose |
| *Tsfa* | TGTGACAGTCAAGGCTCTAATATC | GCTCGTCAGTGATACAGATAGAT | hemicellulose |
| *TsActin* | GGTCAGAAGGATGCCTATGTTG | GGGATTTAGAGGAGCCTCAGTT | Reference gene |
